# Comparability of localization data in transnasal and transoral esophagogastroduodenoscopy

**DOI:** 10.1186/1471-230X-10-116

**Published:** 2010-10-13

**Authors:** Serhat Aymaz, Bernd Krakamp, Oliver Kirschberg, Rolf Lefering

**Affiliations:** 1 Department of Internal Medicine, Holweide Hospital, City of Cologne Hospitals, Ltd., Cologne, Germany; 2 Department of Internal Medicine II, University of Witten/Herdecke, Campus Cologne-Merheim, Germany; 3 Institution for Research in Operative Medicine (IFOM), University of Witten/Herdecke, Campus Cologne-Merheim, Germany

## Abstract

**Background:**

Esophagogastroduodenoscopy is an often-used and safe diagnostic method in gastroenterology. Transnasal esophagogastroduodenoscopy is now an established addition to the endoscopic instrumentarium. Although the two examination methods can be used alongside each other, there is a lack of studies on the comparability of the localization data obtained with the transoral and transnasal methods.

**Methods:**

In 135 adult patients presenting for routine outpatient esophagogastroduodenoscopy, transoral esophagogastroduodenoscopy (TOG) was carried out after transnasal esophagogastroduodenoscopy (TNG), and the distance from the naris or incisors, respectively, to the esophagogastric junction was measured.

**Results:**

The data for 135 patients were analyzed. With the transoral access route, the distance from the upper incisors to the cardia was a mean of 40.5 cm (SD ± 3.4 cm). In the transnasal examinations, the mean distance between the naris and the cardia was 45.6 cm (SD ± 3.5 cm). The correlation analysis showed a very close correlation between the peroral and transnasal data, with a correlation coefficient of *r *= 0.925. On the basis of the regression line calculated using these data, the formula TNG (cm) = 1.1 × TOG (cm) was developed. Using this formula, localization details obtained with one method can be converted into those for the other method.

**Conclusions:**

There is a strong correlation between the localization details obtained with the transnasal and transoral examination methods. The formula for converting localization details from one method to the other, presented here for the first time, is practicable for everyday use and allows rapid conversion.

## Background

Conventional transoral flexible esophagogastroduodenoscopy (EGD) today has an undisputed position as an established and safe method for the endoscopic diagnosis and treatment of diseases of the upper gastrointestinal tract. It is associated with a very low rate of complications of 0.03-0.08% [1,2]. Transoral esophagogastroduodenoscopy (TOG) is usually carried out with intravenous sedation, since manipulation of the hypopharynx causes a gagging reflex that causes discomfort for the patient, makes the examination more difficult, and reduces the acceptability of the examination. Cardiopulmonary complications may occur as adverse side effects of the sedation, and these represent the largest proportion of complications encountered in diagnostic esophagogastroduodenoscopy, at 90% [3-6].

In transnasal esophagogastroduodenoscopy, an ultrathin endoscope is introduced via a naris along the choanae under visual control into the upper gastrointestinal tract, which can be inspected in its entirety. The transnasal access route for esophagogastroduodenoscopy was described sporadically in case reports up to the early 1990 s [5-9]. The first systematic evaluation of transnasal esophagogastroduodenoscopy (TNG) in 1994, in a study including 20 healthy and unsedated volunteers, showed that the method had a high degree of acceptability [10]. Numerous subsequent studies in patients provided positive assessments of TNG with regard to feasibility, safety, acceptance, and the sensitivity and specificity of the findings [11-14]. From the very start, the special aspect of TNG was the good acceptance of the examination method among unsedated patients, since this is associated with a substantial reduction in sedation-related complications and marked cost savings [12].

Transnasal esophagogastroduodenoscopy is now an established and useful addition to the endoscopic instrumentarium. It has proved its value for indications such as difficult transoral access due to previous surgery, trauma, or tumor growth, when there are contraindications to sedatives, to reduce the risk of aspiration, and in particular in follow-up and screening examinations and in outpatients.

Depending on the access route used, data regarding the localization of anatomical structures and endoscopic findings in TOG and TNG are expressed in relation to the distance from the incisors or from the naris, respectively. In view of the anatomical situation, distances obtained with transnasal access can be expected to be longer. Especially in patients with esophageal diseases, e. g. tumor patients with tumors in the upper gastrointestinal tract, the accurate measurement is quite important. There is a lack of studies investigating the comparability of the localization data obtained with the two examination methods when they are used together to complement each other. We therefore carried out a study of consecutive patients with the aim of systematically recording localization details obtained with TOG and TNG and evaluating their comparability.

## Methods

In routine work in our endoscopy department, we use TNG in addition to traditional TOG, depending on the indication and the patient's preferences. A total of 135 consecutive patients presenting for elective outpatient esophagogastroduodenoscopy agreed to take part in the consecutive study. Statistical calculation of the numbers of patients required was carried out on the basis of the conservative assumption of a distribution of the measurements of 3 cm around the mean and an accuracy of ± 1 cm.

After the patients had provided written informed consent to participate in the study, the patient's height was recorded on the way into the examination room, using a measuring tape attached to the door. Routine TNG was carried out with the patient in the left lateral position. Depending on the patient's preferences, local anesthesia of the pharynx and nasal mucosa or intravenous sedation was administered. Local anesthesia of the pharynx and nasal mucosa was carried out with two applications each of a 2% lidocaine spray (Xylocaine^® ^pump spray; AstraZeneca, Ltd., Wedel, Germany) and the application of lidocaine gel (Xylocaine^® ^gel 2%; AstraZeneca) in the naris being intubated. Intravenous sedation was administered with 5 mg midazolam (Dormicum^(r)^; Hoffmann-La Roche, Inc., Grenzach-Wyhlen, Germany), and if necessary with additional administration of propofol 1% (Disoprivan^® ^1%; AstraZeneca). After a complete endoscopic examination of the upper gastrointestinal tract as far as the descending duodenum, the distance from the naris to the cardia was read from the shaft of the device during the withdrawal of the gastroscope. After the examination, the endoscope was advanced perorally to behind the cardia, and after it had been straightened, the distance from the esophagogastric junction to the upper incisors was read from the endoscope shaft. During both measurements, the Z-line or upper margin of the longitudinal gastric folds served as orientation marks.

The study only included adult patients presenting for routine outpatient EGD who consented to undergo TNG after receiving appropriate information. Recruitment of patients for the study was carried out during the information discussion preceding EGD.

Patients who had thrombocytopenia or coagulopathy were excluded from the study. In general, the esophagogastroduodenoscopy was conducted in accordance to the guidelines of the German Society of Digestive Diseases.

All of the examinations were conducted with Fujinon EG470-N video gastroscopes. Documentation of the data was initially carried out using the department's routine documentation sheet. The data were later transferred to Microsoft Excel 2000. Statistical evaluation of the data was carried out using the Statistical Program for the Social Sciences (SPSS), version 10.0.

The study was performed with the approval of the ethics committee of the University of Cologne ("Ethik Kommission der Medizinischen Fakultät der Universität zu Köln", Ref.-no.: 05-003, 21^st ^of February 2005).

## Results

**Figure 1:**
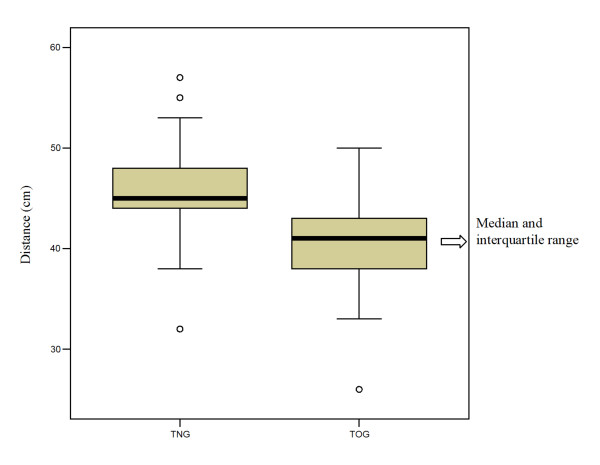
**Box plot illustration of the distance to the cardia (cm) in the transnasal esophagogastroduodenoscopy (TNG) and transoral esophagogastroduodenoscopy (TOG) groups**.

The data for all 135 patients recruited for and examined in the study were evaluated relative to the study aim. With transoral access, the distance from the upper incisors to the cardia was a mean of 40.5 cm (SD ± 3.4 cm), with a range of 26-50 cm. In the transnasal examinations, the mean distance between the naris and the cardia was 45.6 cm (SD ± 3.5 cm), with a maximum of 57 cm and a minimum of 32 cm. The difference in the mean values with TOG and TNG was 5.0 cm (SD ± 1.3 cm; range 0-10 cm) (Table 1). The difference was also confirmed in the comparison of the median values (TOG 41 cm, TNG 45 cm), as the box plot of the values shows (Figure 1).

**Table 1:** Descriptive statistical evaluation (135 patients)

	Minimum [cm]	Maximum [cm]	Mean [cm]	Standard deviation
TNG	32	57	45.6	3.5
TOG	26	50	40.6	3.4
Difference	0	10	5.0	1.3

TNG = transnasal esophagogastroduodenoscopy; TOG = transoral esophagogastroduodenoscopy.

**Figure 2:**
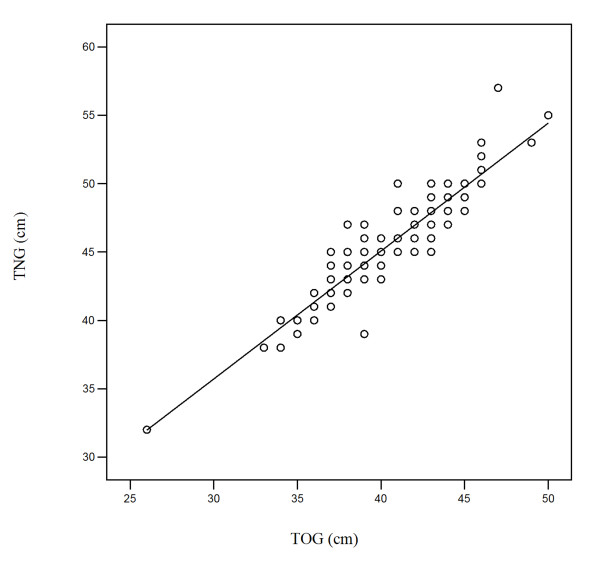
**Graphic depiction of the correlation between measurement points obtained with transoral esophagogastroduodenoscopy (TOG) and transnasal esophagogastroduodenoscopy (TNG): the regression line**. The regression line is described by the equation TOG (cm) = 0.914 × TNG (cm) - 1.115 or after correction as follows TOG (cm) = 0.89 × TNG (cm).

Pearson correlation analysis showed a very close correlation between the transnasally measured values and the values obtained transorally. The analysis showed a correlation coefficient of *r *= 0.925. This represents a specificity measure of *R*^2 ^= 0.85 - i.e., the distribution of the TOG values can be explained by 85% of the TNG values (Figure 2). The regression line is described by the equation TOG (cm) = 0.914 × TNG (cm) - 1.115. After correction of the constant coefficient to 0, the correlation line modified in this way through the zero point is described as follows: TOG (cm) = 0.89 × TNG (cm).

## Discussion

Esophagogastroduodenoscopy is one of the most frequently conducted endoscopic examinations in gastroenterology. Peroral access in the sedated patient is still the standard examination procedure in esophagogastroduodenoscopy [15,16]. Particularly against the background of sedation-associated complications and cost-reduction considerations, esophagogastroduodenoscopy in the unsedated patient via the transnasal access route is becoming increasingly important. As it can be expected that the two methods will be used on a complementary basis alongside each other in the near future, an evaluation of the localization data obtained with each is indispensable in order to allow the findings to be compared. In this consecutive study, an attempt was made to record systematically the localization data obtained with the two methods, to evaluate their comparability and to develop a formula for converting the localization data that is capable of being used in everyday practice.

In agreement with previous reports in the literature the distance from the upper incisors to the cardia in transoral EGD was a mean of 40.5 cm (SD ± 3.4 cm) in the group of patients studied here [17]. The distance from the naris to the cardia during transnasal EGD was measured for the first time here in a larger group of patients. The distance was a mean of 45.6 cm (SD ± 3.5 cm), with a maximum of 57 cm and a minimum of 32 cm. The difference between the mean values in TOG and TNG was 5.04 cm (SD ± 1.3 cm); this was significant and corresponded to the requirement that a longer distance must be present due to anatomical conditions in the transnasal access route.

## Conclusions

The corresponding measurement points in transoral and transnasal esophagogastroduodenoscopy showed a high degree of correlation, with a correlation coefficient of *r *= 0.925. This is the prerequisite for producing a formula on the basis of the regression line in order to calculate the localization data. As a version of the above equation capable of being used in everyday practice, the approximate formulas TOG (cm) = 0.9 × TNG (cm) or TNG (cm) = 1.1 × TOG (cm) can be used. This means that the TNG value is on average one-tenth larger than the TOG value. This provides for the first time a formula with which localization data obtained using TNG or TOG can be intraindividually, reliably, and reproducibly converted into the corresponding values for the alternative method.

## Competing interests

None of the authors received any particular support for this study. The authors have no conflict of interest.

## Authors' contributions

SA and BK conceptualized, designed the study and analyzed the data. OK participated in the analysis and the interpretation and critically revised the manuscript. RL performed the statistical analysis of the data. All authors read and approved the final version of the manuscript.

## Pre-publication history

The pre-publication history for this paper can be accessed here:

http://www.biomedcentral.com/1471-230X/10/116/prepub
